# Career opportunity of whistle-blower in the workplace: the role of privacy legislation and supervisor support

**DOI:** 10.1016/j.heliyon.2022.e10962

**Published:** 2022-10-11

**Authors:** Aden Rosadi, M. Sandi Marta, Dedi Supriadi, Ahmad Sanusi, Yusuf Somawinata

**Affiliations:** a Faculty of Sharia and Law, UIN Sunan Gunung Djati, West Java, Indonesia; b Faculty of Economic and Islamic Business, UIN Sunan Gunung Djati, West Java, Indonesia; c Faculty of Adab and Humanities, UIN Sunan Gunung Djati, West Java, Indonesia; d Faculty of Sharia and Law, Sultan Maulana Hasanuddin State Islamic University of Banten, Indonesia

**Keywords:** Affective commitment, Privacy legislation, Whistle-blowing, Career opportunity, Supervisor support, Government employee

## Abstract

**Purpose:**

This study aims to explain the interaction of perceived privacy legislation on the relationship between affective commitment and whistle-blowing. Additionally, it seeks to determine the consequences of whistle-blowing on employee careers moderated by perceived supervisor support in government organizations.

**Design/methodology/approach:**

A Moderated Regression Analysis was used to analyze survey data from 411 local government employees in West Java, Indonesia.

**Findings:**

The result found a positive relationship between affective commitment and whistle-blowing. Perceived privacy legislation also had a significant moderating effect on the correlation between the two variables. Furthermore, whistle-blowing found a positive relationship with career opportunities, moderated by perceived supervisor support.

**Originality/value:**

The finding may contribute to the extension of scientific literature by making privacy legislation a moderator with the potential to increase employee affective commitment to whistle-blowing behavior. It determines the relationship between whistle-blowing on employee careers opportunity moderated by perceived supervisor support. In contrast, previous studies only focused on factors influencing whistle-blowing behavior.

## 1 Introduction

Many organizations attempt to create a conducive work environment and avoid employee misconduct (Skivenes and Trygstad, 2010; Ugaddan and Park, 2019). In Indonesia, the institutions that have committed the most criminal acts of corruption are local governments, with 210 cases from 2018 to 2020. Meanwhile, other institutions have lower rates than local governments (Bahuri, 2020). The cases were revealed due to several factors, one of which was through the whistle-blowing system, namely the People’s Online Aspiration and Complaints Service (LAPOR) (Soegiono, 2017).

The use of LAPOR to prevent corruption can be found in government agencies. LAPOR is a system created by the central government to accommodate corruption reports from employees of organizations or the public (Ngatikoh et al., 2020). The program is protected by a Law regulating whistle-blower protection, Law Number 7 of 2006, which reported that the State is obliged to provide whistle-blowers legal protection. However, the implementation still has limitations (Juwita, 2016). As a result, the employee is afraid to conduct whistle-blowing (Nawawi and Salin, 2015; Oladinrin et al., 2017; Perks and Smith, 2008) for fear of being considered a troublemaker in the organization.

The results of previous studies stated that whistle-blowing was influenced by transformational-oriented and transactional-oriented leadership (Caillier and Sa, 2016), cultural factors, organizational factors, the materiality of misconduct, individual factors (Alinaghian et al., 2017), person-group fit, and person-supervisor fit (Kyu Wang, et al., 2018), truthful leadership, organizational justice, and motivational factor (Ugaddan and Park, 2019). This research focused mainly on the variables that potentially influence whistle-blowing. However, the consequences have not been discussed, especially regarding the career opportunities of whistle-blowers. An empirical research model is formulated by determining several hypotheses that determine the relationship between affective commitment, whistle-blowing, perceived privacy legislation, perceived supervisor support, and career opportunity to fill the gap. Moreover, this study was conducted in the local government sector, which has many corruption cases and is still rarely studied.

The objectives are to analyze the relationship between affective commitment influencing whistle-blowing attitudes on local government employees and privacy legislation. Second, to analyze the consequences of whistle-blowing behavior on employee career development which is interacted with perceived supervisor support. This study uses survey data on 411 local government employees in West Java to empirically analyze this objective. Furthermore, the data is processed using moderated regression analysis to answer the research hypothesis.

The study is structured as follows: after this introductory perspective, the literature review is presented in Section 2. Section 3 contains a detailed method, followed by the study results in Section 4. Discussion and conclusions are in Section 5, and the implications of the findings are presented in Section 6.

## 2 Literature review

### 2.1 Prosocial behaviour theory

Prosocial Behavior Theory is considered appropriate to complement a study that correlates affective commitment, supervisors' support, whistle-blowing, and the career of employees in the organization to explain the concept. This study is also conducted based on the findings of several previous studies such as (Dozier and Miceli, 1985; Nawawi and Salin, 2015). The theory explains that an employee will try to improve welfare through efforts and actions to maintain the organization. Employees have a high emotional attachment to the organization from the first day of work. Therefore, prosocial behavior has been embedded in employees to form a strong commitment to protecting the organization, and when their position is safe, the level of prosperity will be high. On the other hand, the environment is one of the factors that can weaken or strengthen individual attitudes and behavior. Therefore, the organization should maintain its employees through rules that can improve their welfare.

### 2.2 Affective commitment and whistle-blowing

Commitment is the desire to remain devoted to an organization. Committed individuals bind themselves to groups, organizations, and shared goals. This condition is related to social identity theory, where individuals can be classified into social categories. In other words, this theory explains the social psychological analysis of group membership, group processes, and relationships within groups (Ames et al., 2015). Commitment strongly relates to employee identity (Herrera and De Las Heras-Rosas, 2021). Therefore, a leader should maintain this concept by providing motivation, inspiration, and organizational support (Jaros, 2007). Affective commitment refers to an emotional attachment to identification and involvement in the organization (Meyer and Allen, 1996; Singh et al., 2020). Employees with an effective attitude will reflect their loyalty to the organization and express concern for sustainable success and progress (Somers and Casal, 2007).

Previous studies showed that affective commitment significantly affected attitudes toward whistle-blowing (Alinaghian et al., 2017; Alleyne, 2016; Marta and Eliyana, 2019). Employees with affective commitment will emotionally act for the progress of the organization. In addition, employees with affective commitment will try to protect the organization from the deviant actions of people (Chen, 2019). Affective commitment is an organizational fortress in fostering awareness to conduct whistle-blowing. The higher the affective commitment, the more potential the employee to conduct whistle-blowing (Lavena, 2016; Marta and Eliyana, 2019).

H1a affective commitment has a positive relationship with whistle-blowing behavior.

### 2.3 Affective commitment and whistle-blowing behavior: the role of privacy legislation

There is a long history of developing laws and regulations regarding privacy, hence it should be studied through a different scientific approach (Škrinjaric et al., 2019). Laws and regulations are legal products that regulate individual behavior in a country (Forji, 2010). Furthermore, the law has a large place to regulate the public or private sector in conducting the organization, including the application of privacy legislation in companies.

The concept of perceived privacy legislation in management science begins with marketing research, which emphasizes rights protection (Boerman et al., 2018; Xu et al., 2011). Privacy is an individual, personal right of philosophical and moral origins (Johansen et al., 2022; Mitrou and Karyda, 2006). The legislation relates to belief that organizations will protect employees' privacy through laws and regulations. The form is evidenced by institutional structural guarantees provided by government agencies (Wang, 2019).

In the organization, perceived privacy legislation is the basis to conduct whistle-blowing, especially among employees with high affective commitment. According to the study by Near and Miceli (1996), whistle-blowing behavior was influenced by the interaction between individual factors, namely affective commitment, and organizational factors. Likewise, environmental factors include privacy legislation and the game’s rules in organizations related to whistle-blowing. The results of Ugowe and Adebayo (2018) reinforced that a whistle-blower had to be protected by legislation to increase braveness, tell the truth, and be protected from retaliation. The finding is supported by Bakirci (2019), who stated that whistle-blowing requires proper protection through legislation to protect the whistle-blower.

Implementing regulations in the organization determines the joint commitment of the leaders and employees to implementing the whistle-blowing system. In other words, affective commitment can maximally influence the whistle-blowing behavior in the organization when it is encouraged by the support of a formally established safety, welfare, and security guarantee system.

H1b Perceived privacy legislation will moderate the relationship between affective commitment and employees' whistle-blowing behavior.

### 2.4 Whistle-blowing behavior and career opportunity

In the view of employees, a career is a goal that should be achieved with the right plan. Neglecting aspects of career planning results in a lack of clarity about the intended career path, resulting in professional obstacles. Employee career development depends on planning made by the organization. A career is the sequence of a person’s work experience over time. Meanwhile, career opportunity is defined as perceptions of the degree to which work assignments and job opportunities are available within their current organization (Kraimer et al., 2011). Another opinion regarding career opportunity defines it as the knowledge that today’s work will enhance the person’s value in future opportunities (Chay and Aryee, 1999; Huo, 2021).

A career can be achieved quickly through heroic behavior in maintaining the organization’s continuity. Heroic behavior signifies an employee with loyalty to the organization (Kinsella et al., 2019). The form of loyalty is implemented to protect the organization from the actions of irresponsible that may damage the organization. In other words, an employee conducts whistle-blowing for the person in charge of the organization, namely the supervisor. Whistle-blowing action to maintain the organization is very necessary to the supervisors. Therefore, whistle-blowers’ careers should be supported and developed. According to social exchange theory, the interaction relationships in organizations have rewards, sacrifices, and benefits that complement and influence each other (Cropanzano and Mitchell, 2005; Raj, 2021). Furthermore, employees will be rewarded after making sacrifices in the organization. Rewards and benefits will be low when they are not willing to be devoted. Whistle-blowing behavior is a sacrifice expected to obtain rewards from the organization.

A previous study stated that whistle-blowing affected employees in the organization (Aslam et al., 2021; Delk, 2013). Supervisors, colleagues, and the organization can also feel the significant influence. However, whistle-blowers should consider all the conditions before they conduct the action. Another study stated that the tendency to perform whistle-blowing had an impact on meeting the need for rewards within the organization (Alinaghian et al., 2017; Wise, 1995). The reward is one of the needs to be achieved in supporting the success of the employees.

H2a Whistle-blowing behavior has a positive relationship with employee career opportunities.

### 2.5 Whistle-blowing behavior and career opportunity: the role of supervisor support

The effect of whistle-blowing on an employee’s career cannot be predicted, hence proper planning should be conducted. This condition should be neutralized through the supervisor’s support, who directly influences employees (Near and Miceli, 1996). Supervisors have a vital role in influencing attitudes, expectations, and behavior (Kalliath et al., 2020). According to Hsu (2011), perceived support is employees' perception of their relationship with supervisors and how they pay attention to interests. Another opinion relates to the perceptions that supervisors value their contribution and welfare (Arici, 2018).

This is related to the level and quality of support provided in carrying out the organization’s work. In this perception, an assessment is formed to offer emotional, informational, and material support for organizational progress (Suan and Nasurdin, 2016). Furthermore, the support can also provide continuous protection when whistle-blowing is conducted to guarantee job career development. A study on hotel employees at Cameron found that support from the organization, especially from the supervisors, was evidenced to assist employees' careers (Choi et al., 2012). The finding is also supported by Yang et al. (2018), where supervisor support influenced employee careers. Therefore, supervisors are obliged to maintain the security of employees to report harmful behavior towards the organization. It may be tough to locate suitable successors because they are the firm’s most important asset. Therefore, attention and support should be provided morally and materially.

H2b Perceived supervisor support will moderate the relationship between whistle-blowing and employee career opportunity.

**Figure 1:**
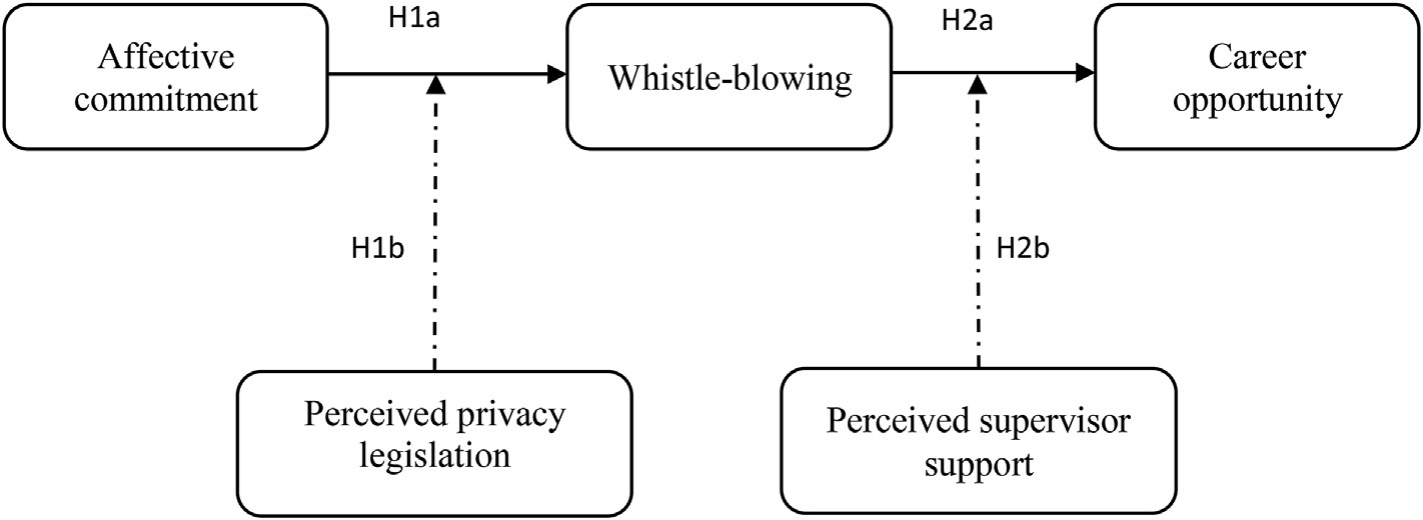
Conceptual model.

Figure 1 explains the conceptual model on the relationship between affective commitment and whistle-blowing moderated by perceived privacy legislation and the interaction of supervisors' support.

## 3 Methodology

### 3.1 Participants and procedures

The data survey was collected through a questionnaire administered to employees in May and June 2021. This was conducted using a simple random sampling technique on 500 employees in 8 local governments in West Java, Indonesia, provided the respondents had a minimum of 1 year. Before data collection, this study followed the ethical considerations mandated by the Research Ethics Committee at UIN Sunan Gunung Djati Bandung in Indonesia, and the necessary ethical approvals were obtained.

Questionnaires were distributed during working hours to provide a cover letter explaining the objectives to the respondents, and assured them that the results would remain confidential. Therefore, all respondents agreed to participate and fill out the questionnaire.

At the end of June, 421 questionnaires were returned with a response rate of 84.2% from a total of 500 distributed, and only 411 (82.2%) met the criteria for analysis. The sample consisted of 237 or 58% males and 174 or 42% females, and most of the participants were in a partnered relationship (married or cohabitating), with 319 or 77.7%, and 92 or 22.39% being single or separated, and divorced or widowed. Furthermore, 211 or 51%, 153 or 37%, 47 or 11% of respondents were between 20 and 29 years old, 30–39, and more than 40, respectively. Regarding academic qualification, 45 had a college degree or below, 277 had an undergraduate degree, and 89 had a graduate degree or higher, representing 11%, 67%, and 22% of the sample.

### 3.2 Measurement

All variables were quantified on a 5-point Likert scale from “strongly disagree = 1” to “strongly agree = 5”. The details of measurement of each construct are as follows:

The affective commitment was measured with the 8-item scale as referred to in (Jaros, 2007; Meyer et al., 1993; Allen and Meyer, 1990). Sample items from this scale included, “I would be very happy to spend the rest of my career with this organization.” Perceived privacy legislation was modified from 3-item scales obtained from the study (Bakirci, 2019; Wang, 2019; Xu et al., 2011). Sample items from this scale included, “With the privacy protection laws, I believe that my personal information will be kept private and confidential when reporting wrongdoing.”

Whistle-blowing behavior was measured with the 6-item. Furthermore, 5-items were developed by reference to Nayir and Herzig (2012). Sample items from this scale include, “I would report the wrongdoing to the appropriate persons outside the workplace.” One item was developed by reference (Rustiarini and Sunarsih, 2017). Sample item “I would report fraudulent activity to the appropriate persons within the workplace.” Perceived supervisor support was modified from 5-item scales obtained from the study (Karasek et al., 1982). Sample items include the encouragement to exchange opinions and ideas about whistle-blowing. Career opportunity was measured with the 3-item scale that Kraimer et al. (2011) developed. Meanwhile, sample items from this scale include, “There are career opportunities within the organization that are attractive to me.”

### 3.3 Data analysis

The Moderated Regression Analysis (MRA) was applied to test the study hypothesis. This statistical analysis tries to analyze and explain the moderating variable’s position in the study (Sharma et al., 1981). Additionally, the test intends to determine the moderating variable’s role in the relationship between the independent and dependent variables (Helm and Mark, 2012). STATA 13 software Copyright 1985–2013 StataCorp LP was used to simplify the statistical analysis of Moderated Regression Analysis.

## 4 Results and analysis

Before analyzing the data, the validity and reliability tests were applied to the measures used towards the variables (Ferdinand, 2014; Hair et al., 2013; Sekarandan, 2009). Table 1 showed that the confirmatory value of factor analysis had a loading factor value of >0.5 (Gürlek, 2020). The value ranged from 0.549 to 0.894, which showed satisfactory convergent validity. Likewise, the composite reliability value was >0.7 (Fornell and Larker, 1981). This is also supported by Cronbach’s alpha of >0.6 (Hair et al., 2013)

**Table 1:** Validity and reliability test.

No	Variables	Item	Loading (ʎ)	CR	AVE	Alpha
1	Affective commitment	AC1	0.742	0.909	0.560	0.784
2		AC2	0.781			
3		AC3	0.855			
4		AC4	0.734			
5		AC5	0.800			
6		AC6	0.747			
7		AC7	0.549			
8		AC8	0.741			
9	Perceived privacy legislation	PPL1	0.769	0.843	0.642	0.817
10		PPL2	0.823			
11		PPL3	0.811			
12	Whistle-blowing	WB1	0.756	0.892	0.587	0.871
13		WB2	0.577			
14		WB3	0.635			
15		WB4	0.894			
16		WB5	0.815			
17		WB6	0.867			
18	Perceived supervisor support	PPS1	0.550	0.849	0.535	0.741
19		PPS2	0.783			
20		PPS3	0.792			
21		PPS4	0.769			
22		PPS5	0.734			
23	Career opportunity	CO1	0.761	0.772	0.536	0.698
24		CO2	0.576			
25		CO3	0.835			

The correlations, discriminant validity, and descriptive statistics of the variables are shown in Table 2. Meanwhile, Tables 3 and 4 show the regression results.

**Table 2:** Mean, standard deviation, correlation, and discriminant validity.

Variables	Mean	Std. Dev.	1	2	3	4	5
Affective commitment	4.2213	0.53636	0.748				
Perceived privacy legislation	4.1365	0.71297	.180∗∗	0.801			
Perceived supervisor support	4.2990	0.58660	.398∗∗	.129∗∗	0.731		
Whistle-blowing	4.1040	0.67357	.628∗∗	.218∗∗	.447∗∗	0.766	
Career opportunity	4.2211	0.61218	.309∗∗	0.086	.297∗∗	.247∗∗	0.732

N = 411; ∗p < 0.05; ∗∗p < 0.01, two-tailed test.

**Table 3:** Summary of moderated regression analysis for perceived privacy legislation as a moderator of affective commitment and whistle-blowing.

Variables	Model 1		Model 2		Model 3	
	Coef.	P	Coef.	P	Coef.	P
Constant (a)	0.773	0.000	0.450	0.631	2.750	0.020
Affective commitment	0.789	0.000	0.764	0.000	0.209	0.459
Perceived privacy legislation			0.102	0.005	-0.455	0.108
Affective commitment x perceived privacy legislation					0.134	0.047
F test	266.20	0.000	139.28	0.000	94.85	0.000
R Square	0.395		0.406		0.412	
Adjusted Square	0.393		0.403		0.407	

N = 411; ∗p < 0.05; ∗∗p < 0.01.

**Table 4:** Summary of moderated regression analysis for perceived supervisor support as a moderator of whistle-blowing and career opportunity.

Variables	Model 1		Model 2		Model 3	
	Coef.	P	Coef.	P	Coef.	P
Constant (a)	3.300	0.000	2.645	0.000	7.427	0.000
Whistle-blowing	0.225	0.000	0.129	0.007	-1.117	0.002
Perceived supervisor support			0.242	0.000	0.286	0.000
Whistle-blowing x perceived supervisor support					0.286	0.000
F test	26.46	0.000	23.68	0.000	21.68	0.000
R Square	0.060		0.104		0.138	
Adjusted Square	0.059		0.099		0.131	

N = 411; ∗p < 0.05; ∗∗p < 0.01.

The mean, standard deviation, correlation, and discriminant validity of variables are reported in Table 2. The results showed that all direct correlations were significant. Specifically, “affective commitment” was positively correlated with whistle-blowing among the local government employees in West Java (r = 0.628, p < 0.000). “Perceived privacy legislation” was positively correlated with whistle-blowing among the local government employees (r = 0.218, p < 0.000). Perceived supervisor support was positively correlated with career opportunities (r = 0.297, p < 0.000). Whistle-blowing was positively correlated with employee career opportunities (r = 0.247, p < 0.000). These findings provided general support for all hypotheses. Based on the discriminant validity test, it can be stated that the root value of the AVE is higher than the correlation value between different variables (Fornell and Larker, 1981). Therefore, the study instrument used met the requirements to examine the correlation between affective commitment, perceived privacy legislation, perceived supervisor support, whistle-blowing, and career opportunity.

Moderated Regression Analysis, as presented in Table 3, was applied to test hypotheses 1a and 1b. Model 1 showed that hypothesis 1a, “affective commitment,” had a positive relationship with whistle-blowing among the local government employees in West Java (β_1_ = 0.789, p < 0.000). Model 2 showed that perceived privacy legislation positively impacted employee whistle-blowing (β_2_ = 0.102, p < 0.000), and moderating variable β_2_ showed a significant value. In Model 3, hypothesis 1b showed that the effect of “affective commitment” was moderated by perceived privacy legislation among the employees (β_3_ = 0.134, p < 0.047). The model was able to account for 41.20% of the variance, F = 94.85, p < 0.000, R2 = 0.412. The hypothesis 1b analysis result indicated that the value of moderating variable β_3_ was significant.

The Moderated Regression Analysis test showed that β_1,_ β_2,_ and β_3_ were significant. Hence, it can be concluded that perceived privacy legislation was a quasi-moderator in the correlation between the “affective commitment” and whistle-blowing among employees (Sharma et al., 1981).

As indicated in Table 4, moderated regression analysis was used to test hypotheses 2a and 2b. Model 1 showed that hypothesis 2a had a positive relationship with career opportunities (β_1_ = 0.225, p < 0.000). The result showed that the value of β_1_ was significant. Model 2 showed that perceived supervisor support positively impacted career opportunities among the local government employees in West Java (β_2_ = 0.242, p < 0.000). The value of the moderating variable β_2_ was significant. In Model 3, hypothesis 2b showed that the effect of whistle-blowing on career opportunity was moderated by perceived supervisor support (β_3_ = 0.286, p < 0.000). Meanwhile, it was able to account for 13.80% of the variance, F = 21.68, p < .000, R2 = 0.138. The result of hypothesis 2b analysis indicated that the value of β_3_ was significant. The moderated regression analysis test showed that β_1,_ β_2,_ and β_3_ were significant. Hence, perceived supervisor support was the quasi-moderating variable in the correlation between whistle-blowing and career opportunity (Sharma et al., 1981).

## 5 Discussion and conclusion

Employees in the organization should keep and maintain the organization to ensure growth. They are also obliged to protect the organization from existing problems, such as the criminal act of corruption conducted by supervisors and colleagues. Therefore, organizations should find solutions to create initial protection to avoid corrupt behavior. The whistle-blowing system can prevent corrupt behavior, where employees are the key actors in the mechanism (Alinaghian et al., 2017; Jalilvand et al., 2017).

The results of the analysis for hypothesis 1a found that affective commitment had a positive relationship with whistle-blowing. An employee with a high affective commitment is responsible for protecting the organization from internal and external problems (Wiisak et al., 2022). In Indonesia, local governments always try to instill an affective commitment during work orientation. This orientation provides an understanding that an employee should be strongly concerned, including caring about harmful actions toward the organization’s reputation. Employees also should protect the organization from corrupt people.

The relationship between affective commitment and whistle-blowing is supported by prosocial behavior theory (Dozier and Miceli, 2011; Nawawi and Salin, 2015). This theory states that an organization member has a sense of attachment to maintain and improve welfare (Ahmad et al., 2014; Kang, 2022). Moreover, the action of whistle-blowing is an example of prosocial behavior based on the desire to guard the organization from misconduct. Hence, it is clear that the relationship between affective commitment and whistle-blowing is a part of prosocial behavior (Nawawi and Salin, 2015).

Hypothesis 1b states that the correlation between affective commitment and whistle-blowing is moderated by perceived privacy legislation, which is a rule made to protect whistle-blowing. This rule is a form of organizational concern in maintaining employee honesty. One of the rules that protect whistle-blowing in local governments is Law Number 7 of 2006, which states that the State is obliged to provide legal protection for whistle-blowers (Juwita, 2016). The impact of this privacy legislation encouraged self-confidence, which triggered affective commitment to whistle-blowing. Therefore, privacy legislation is crucial to creating the mindset of employees in the organization (Brink et al., 2021). The results of Ugowe and Adebayo (2018) indicated that a whistle-blower had to be protected by legislation, to tell the truth, and be protected from retaliation. Bakirci (2019) also confirmed that whistle-blowing requires proper protection through legislation that can protect the reporter.

The interaction relationship between affective commitment, privacy legislation, and whistle-blowing is supported by attribution theory. This theory states that a behavior is formed by combining internal and external forces. Internal forces are related to factors from within the individual, such as ability, knowledge, and effort. In contrast, external forces are related to factors from outside, such as the environment, opportunities, and rules (Lei et al., 2021; Xu et al., 2011). Affective commitment and privacy legislation are internal and external forces for the employees. These forces define the whistle-blowing behavior, which is also reinforced by the social exchange theory, in which the value shapes individual behavior (Cropanzano and Mitchell, 2005). Privacy legislation is a product created by an organization to protect employees who conduct whistle-blowing. In other words, employees safeguarded by privacy laws will feel supported by their employers and perform as expected (Park et al., 2018).

Hypothesis 2a states that whistle-blowing statistically has a positive relationship with career opportunities. A career is very important because it improves welfare (Huo, 2021). Each employee will compete to obtain a different career (Lei et al., 2021), which can be achieved through the whistle-blowing path. This pathway emphasizes actions to maintain organizational security from actions that can damage the organization. In other words, whistle-blowing have the supervisor’s support to be promoted to save the organization. This statement is supported by regulatory focus theory, that an individual has two strategies in achieving goals (career), namely focusing on promotion and prevention (Ahn et al., 2020; Gürlek, 2020). Individuals who focus on promotion tend to plan in various ways, while those focused on prevention are more likely to maintain organizational safety and avoid risks. Individuals focusing on prevention can advance their careers in the organization through whistle-blowing actions (Bishop-Monroe et al., 2021; Heese and Pérez-Cavazos, 2021). This theory is also strengthened by a previous study, where whistle-blowing significantly affected employees in the organization. This effect is also experienced by supervisors, colleagues, and organizations (Delk, 2013).

Hypothesis 2b states that the correlation between whistle-blowing and career opportunity is moderated by perceived supervisor support. Employee careers and supervisor support are closely related, hence, a different approach is needed to obtain both (Kohlmeyer et al., 2017; Kundi et al., 2021). However, when whistle-blowers do not have supervisors' support, they become troublemakers and can narrow their promotions and salary (Nawawi and Salin, 2015; Oladinrin et al., 2017). Therefore, they should synergized to have the same opportunity and a high priority in career opportunities.

Several local governments implement whistle-blowing regulations for those who report criminal acts of corruption (Inawati and Sabila, 2021). The action supported by the leader policy can guarantee the welfare of the reporter. Furthermore, supervisor support can strengthen the relationship between whistle-blowing and employee career. Whistle-blowing is an act of employees to protect the organization from the behavior of the damaged aspects.

The way employees maintain the organization by whistle-blowing is a form of prevention from wrongdoings. Therefore, such action is included in the regulatory focus theory, and whistle-blowing is a tool to protect the organization from damage actions. Furthermore, supervisor support is a supporting factor to keep employees and provide a better career. It is a very important factor in encouraging employee career advancement and can form the impression that supervisors care about their well-being and contributions (Tahiry and Ekmekcioglu, 2022; Yang et al., 2018). Consequently, this support is considered the most effective way to manage subordinates' attitudes (Richardson, 2022; Yang et al., 2018).

In conclusion, this study explains in-depth the perceived privacy legislation’s role in the relationship between affective commitment and whistle-blowing. The findings showed that affective commitment and whistle-blowing have a positive relationship and are strengthened by perceived privacy legislation. In other words, employees with high affective commitment had a great opportunity to conduct whistle-blowing under the support of privacy legislation. Furthermore, this study also found that whistle-blowing positively impacted career opportunities in local government organizations, and the relationship is strengthened by perceived supervisor support. Employees brave enough to do whistle-blowing had a great opportunity to achieve a career in government organizations when they provided extra employee support (Lavena, 2016).

Finally, this study assumes that whistle-blowing in local government can be the most effective control media to safeguard the organization from damage actions (Alinaghian et al., 2017; Bishop-Monroe et al., 2021). In other words, whistle-blowing is the right method to prevent violations, fraud, and abuse of power in local government (Jalilvand et al., 2017). Employee whistle-blowing can be increased through affective commitment (Alleyne, 2016; Marta and Eliyana, 2019) and legal protection. Additionally, perceived supervisor support drives the employees to conduct whistle-blowing. This support is based on mutual trust between employees and supervisors (Lavena, 2016).

## 6 Implications of findings

### 6.1 Theoretical implications

The findings provide theoretical implications for implementing whistle-blowing in the context of Indonesian local government employees. Affective commitment is positively related to whistle-blowing, and the relationship is moderated by perceived privacy legislation. The findings are useful in observing the development of whistle-blowing in local governments. Theoretically, this study tries to explain the relationship of whistle-blowing to the career opportunities of local government employees. Research with a quantitative approach on the relationship between whistle-blowing and career opportunity is rarely conducted. Previously, research on whistle-blowing was performed with a qualitative approach (Bakirci, 2019; Nwoke, 2019; Ugowe and Adebayo, 2018). The findings contribute to the management literature and enrich the depth of the existing literature through empirical confirmation with the context of developing countries.

### 6.2 Practical implications

This study contributes to government organizations regarding the implementation of the whistle-blowing system by local government employees in West Java. Whistle-blowing ensures the long-term viability of a government institution, but only when a pleasant work atmosphere is developed and maintained (Alleyne, 2016). Besides, the government is expected to create a whistle-blowing mechanism that protects the whistle-blower’s identity, accompanied by clear standard operating procedures (Bakirci, 2019; Ugowe and Adebayo, 2018). This mechanism can use electronic reporting media, where employees can hide their identities when reporting, provided the report is accompanied by concrete evidence. To avoid retaliation from reported employees, organizations should use a mutation system for employees who commit malpractice (Oladinrin et al., 2017). This system can keep the reporter away from retaliating behavior. Subsequently, they feel protected and secured by the system created by the government. Additionally, the government should provide warnings and guidance for employees who commit malpractice to learn from their wrongdoings.

Local governments should create a career advancement model for employees who conduct whistle-blowing by considering the aspects of the affective commitment (Lavena, 2016). Career advancement is a form of organizational concern for themselves and other employees. In addition, the government should guide supervisors to always support their subordinates in whistle-blowing (Dai et al., 2016). The model should be packaged into an organizational mechanism that continuously encourages employees to testify about malpractice behavior (Dawley et al., 2010). Furthermore, government organizations are expected to provide knowledge through education and training strategy mechanisms. This supports a vigilance culture during meetings, pre-work briefings, email alerts, formal training, and creating warning boards.

### 6.3 Limitations and future directions

This study has several limitations to be addressed. First, it was conducted at local government organizations in Indonesia. Consequently, the findings may not apply to other countries. Therefore, further studies are expected to include samples from foreign local government organizations, such as Indonesia vs Singapore or Indonesia vs Malaysia. Second, it is only focused on perceived supervisor support as a moderator. Supervisory political support with organizational and political climate as moderators should be considered in the relationship between whistle-blowing and career opportunity. Third, moderation analysis was only used to analyze the relationship between variables. Further studies should use mediation analysis to analyze the simultaneous relationship between affective commitment, whistle-blowing, and career opportunity. Fourth, the phenomena of affective commitment, perceived supervisor support, whistle-blowing, and career opportunity were explained using data collection survey techniques. These variables should be collected and analyzed in future studies using experimental methods.

## Declarations

### Author contribution statement

Aden Rosadi, Dedi supriadi: Conceived and designed the experiments; Performed the experiments.

Ahmad Sanusi, Yusuf Somawinata: Analyzed and interpreted the data.

M Sandi Marta: Contributed reagents, materials, analysis tools or data; Wrote the paper.

### Funding statement

This research did not receive any specific grant from funding agencies in the public, commercial, or not-for-profit sectors.

### Data availability statement

Data will be made available on request.

### Declaration of interest’s statement

The authors declare no conflict of interest.

### Additional information

Supplementary content related to this article has been published online at [URL].

## Appendix A Supplementary data

measurement.docx measurement.docx
