# Supplementary material for: Career opportunity of whistle-blower in the workplace: the role of privacy legislation and supervisor support
Source: Heliyon. 2022 Oct 11;8(10):e10962. doi: 10.1016/j.heliyon.2022.e10962 (PMC9568826; doi:10.1016/j.heliyon.2022.e10962)
Supplement: measurement.docx [file mmc1.docx]

**Appendix. I**

**Measurement**

**Affective commitment**

1. I would be very happy to spend the rest of my career with this organization.
2. I enjoy discussing about my organization with people outside it.
3. I really feel as if this organization’s problems are my own.
4. I think that I could easily become as attached to another organization as I am to this one.(R)
5. I do not feel like ‘part of the family’ at my organization.(R)
6. I do not feel ‘emotionally attached’ to this organization.(R)
7. This organization has a great deal of personal meaning for me.
8. I do not feel a ‘strong’ sense of belonging to my organization.(R)

**Privacy legislation**

1. I feel confident that privacy protection laws reflect their commitment to protecting my personal information when reporting wrongdoing.
2. With the privacy protection laws, I believe that my personal information will be kept private and confidential when reporting wrongdoing.
3. I believe that the privacy protection laws are an effective way to protect my personal information when reporting wrongdoing

**Whistle-blowing**

1. I would report it using an assumed name
2. I would report the wrongdoing but wouldn’t give any information about myself
3. I would report the wrongdoing to the appropriate authorities outside of the workplace
4. I would provide information to outside agencies
5. I would inform the public of it
6. I report fraudulent activity to the appropriate persons within the workplace

**Supervisor support**

1. Supervisor encourages those he supervises to develop new ways of doing whistle-blowing.
2. Supervisor shows you how to whistle-blowing.
3. Supervisor encourages those he supervises to whistle-blowing.
4. Supervisor offers new ideas for reporting wrongdoing.
5. Supervisor encourages those he supervises to exchange opinions and ideas about whistle-blowing

**Career opportunity**

1. There are career opportunities within the organization that are attractive to me.
2. There are job opportunities available within the organization that are of interest to me.
3. The organization offers many job opportunities that match my career goals.
